# Root Plasticity of *Populus euphratica* Seedlings in Response to Different Water Table Depths and Contrasting Sediment Types

**DOI:** 10.1371/journal.pone.0118691

**Published:** 2015-03-05

**Authors:** Lijuan Wang, Chengyi Zhao, Jun Li, Zhihui Liu, Jianghong Wang

**Affiliations:** 1 State Key Laboratory of Desert and Oasis Ecology, Xinjiang Institute of Ecology and Geography, Chinese Academy of Sciences, Urumqi, Xinjiang, China; 2 College of Resource and Environment Sciences, Xinjiang University, Urumqi, Xinjiang, China; 3 University of Chinese Academy of Sciences, Beijing, China; 4 Bayingol Mongolian autonomous prefecture Environmental Monitoring station, Korla, Xinjiang, China; Pennsylvania State University, UNITED STATES

## Abstract

Riparian plants in arid regions face a highly variable water environment controlled by hydrological processes. To understand whether riparian plants adapt to such environments through plastic responses, we compared the root traits, biomass allocation and growth of *Populus euphratica* Oliv. Seedlings grown in lysimeters filled with clay or clay/river sand sediments under inundation and varying water table conditions. We hypothesized that adaptive phenotypic plasticity is likely to develop or be advantageous in seedlings of this species to allow them to adapt desert floodplain environments. Growth was significantly reduced by inundation. However, rather than following relatively fixed trait and allocation patterns, the seedlings displayed adaptive mechanisms involving the development of adventitious roots to enhance plant stability and obtain oxygen, together with a lower proportion of root biomass. At the whole-plant level, at deeper water table depths, seedlings allocated more biomass to the roots, and total root length increased with decreasing water table depths, regardless of the sediment, consistent with optimal partitioning theory. The sediment type had a significant effect on seedling root traits. *P. euphratica* displayed very different root traits in different sediment types under the same hydrological conditions, showing a greater first-order root number in clay sediment under shallower water table conditions, whereas rooting depth was greater in clay/river sand sediment under deep water table conditions. In clay sediment, seedlings responded to lower water availability via greater root elongation, while the root surface area was increased through increasing the total root length in clay/river sand sediment, suggesting that seedlings facing deeper water tables are not always likely to increase their root surface area to obtain more water. Our results indicate that *P. euphratica* seedlings are able to adapt to a range of water table conditions through plastic responses in root traits and biomass allocation.

## Introduction

Desert floodplains are characterized by extreme temporal heterogeneity in hydrological variability, which is widely considered a major ecological and evolutionary driver in these environments [1–3]. Plants inhabiting such areas must accommodate a wide range of hydrological conditions. In most cases, only a few woody species occupy these complex habitats [3]. Seedling responses to hydrological fluctuations are important in determining the distributions of woody species in these environments, as juvenile plants are usually more susceptible to environmental changes [4–6]. Hence, high mortality often occurs in the juvenile stage [6–8], and recruitment success therefore depends on the ability of seedlings to cope with such environments [8]. Most riparian species are phreatophytes, which are highly dependent on groundwater, and access to groundwater by their roots is essential for seedling establishment as well as sustaining whole-plant growth [6, 9, 10]. However, how the root systems of riparian plants at the seedling stage develop in response to hydrological fluctuations has not been fully elucidated.

Plants in variable habitats are frequently linked to phenotypic plasticity and adaptive plasticity, i.e., environmentally induced phenotypic variation originating from direct selection [3, 11], which is considered to be a major evolutionary response to temporal environmental heterogeneity [11, 12]. Plastic responses to environmental heterogeneity may also involve adaptive adjustments in biomass partitioning at the whole-plant level that reflect variations in resource limitation [13–15]. Although research has shown that adaptive phenotypic plasticity is likely to involve significant costs or functional trade-offs [3, 11, 16–19], it is widely accepted that adaptive benefits of plastic traits as “buffers against spatial or temporal variability in habitat conditions” and a “means of optimizing the acquisition and use of resources” [20–22]. Examples of such benefits include the flooding-induced formation of aerenchyma and adventitious roots, facilitating the internal diffusion of gases [23], and flooding-induced elongation of shoot organs such as petioles and internodes [19, 24, 25], which helps to ameliorate or avoid the adverse effects of flooding.

Given the complex and unpredictable nature of desert floodplains, we hypothesize that adaptive phenotypic plasticity is likely to have developed or be advantageous for seedlings of woody plants in these environments. Rather than following relatively fixed developmental trajectories and allocation patterns, seedlings of dominant woody species in desert floodplains might be expected to invest in the capacity to interpret and respond plastically to hydrological fluctuations (e.g., altering biomass allocation patterns) [9], showing variation in morphological traits, such as adventitious or secondary roots [23], which all aid in restoring the supply of oxygen to the submerged plant tissues. Optimal partitioning of root biomass in response to deeper water tables, for instance, may increase water availability for plants.

*Populus euphratica* Oliv. dominates riparian zones in arid central Eurasia [26, 27], where it plays an important role in stabilizing these vulnerable ecosystems [26, 28]. At present, the world’s largest *P. euphratica* forests occur along the Tarim River and its tributaries in the Tarim basin [28, 29]. As a consequence of land opening campaigns, large areas of *P. euphratica* forests were destroyed after the 1950s [28]. Due to excessive use of water for irrigation, the remaining *P. euphratica* forests are under severe threat [28, 30, 31], and the restoration of these riparian forest ecosystems is of worldwide significance [28]. In these regions, groundwater table depth and flooding can influence the fine-root growth and mortality of *Populus* species [9, 10, 26, 32–34]. Therefore, the response of root traits to varying water availability is expected to be a key aspect explaining recruitment success in riparian ecosystems.

Here, we investigated the root traits of *P. euphratica* seedlings grown in a range of riparian hydrological conditions simulated using lysimeters. We sought to evaluate the adaptive phenotypic plasticity of the root traits of *P. euphratica* seedlings in relation to the highly variable water environments where they become established. Because this species occurs in a variety of floodplain soils [28, 35], we also considered whether the response of root traits to hydrological conditions would be modified by the sediment type.

## Materials and Methods

### Ethics approval

All seeds were collected at random in a *P. euphratica* population of a natural Populus Forest (40°26′58.02″- 40°27′47.09″N, 80°45′38.35″- 80°52′09.31″ E; Altitude, 1005 m) located along the Tarim River. These trees grow in public area where no permission for collection of seeds is needed in China. The experiments were conducted in the Aksu Water Balance Station, Chinese Academy of Sciences (40°37 ′ N, 80°49 ′ E and Altitude: 1031 m, hereafter Aksu Station). The Aksu Station is owned and managed by the Xinjiang Institute of Ecology and Geography Chinese Academy of Sciences, with the aim to study ecology of this area. *P. euphratica* is a protected species in China, but a specific permit for scientific research is not required.

### Plant materials and experimental facility

We grew *P. euphratica* from seeds planted in trays of damp soil in a nursery at the Aksu Station, during September 2011. After nine months of growth in damp conditions, two hundred seedlings 5.0 ± 1.5 cm in height and 0.7 ± 0.2 mm in above-ground diameter were selected and transplanted in individual lysimeters, designed to simulate the hydrological conditions of the riparian floodplain. Each lysimeter consisted of a 1.2 m tall × 0.4 m inner diameter PVC pipe with a drain valve at the bottom and a discharge valve placed at a specific height for water table depth control. During the experiment, when the drain valves were open, water flowed freely out of the lysimeters. To impose the water table treatments, the drain valve was closed, and water was added to the lysimeters through a 2 cm-diameter pipe inserted vertically just above the bottom of the lysimeter so that the lysimeters could be watered from the bottom up [27]. A 10 cm layer of gravel was placed in the bottom of each lysimeter, followed by 90 cm layer clay (0.11 m^3^) collected from the field site in half of the lysimeters and a mixture of 50% clay and 50% river sand in the remaining lysimeters. Seedling heights, tap-root depths and first-order root numbers were recorded for all seedlings prior to transplantation. An additional 10 seedlings that met the above-mentioned size requirements were randomly harvested to obtain initial measurements of growth variables and selected biomass. Five seedling lysimeters of each sediment type were then placed in each of 20 plots and kept moist with freshwater using a hose for a period of one week to allow seedlings to acclimatize before commencing watering treatments. No fertilizer was added to the lysimeters during the experiment because low nutrient availability typically occurs in Tarim riparian zones [27, 36].

### Experimental design

The experiment was conducted over a period of 75 days, from 15 July to 30 September of 2012, during which the daily minimum and maximum temperatures averaged 17.8°C and 37.4°C, respectively. Lysimeters were randomly placed in outdoor trenches. We used a 50% transparent net to ensure the survival of seedlings for the first month after transplantation, after which the seedlings were subjected to full sunlight from 1 July onward. Styrol boards were placed on the south side of each lysimeter to avoid over-heating. A rainfall shelter was used to exclude the confounding effects of rainfall on the lysimeter hydrological treatments, even though Aksu Station exhibits a mean annual precipitation < 50 mm [27].

At the commencement of the experiment, the lysimeters in each plot were randomly assigned to one of four watering treatments: a constant water table of 30, 50 or 70 cm or inundation (5 cm above the soil surface in the lysimeters). During the treatment period, water was added every afternoon (6–8 p.m. local time). For the inundation treatment, the water depth above the soil surface within the lysimeters was kept at > 5 cm, varying from 5 to 7 cm as a result of evapotranspiration. The indicated water levels were maintained throughout the experiment. We harvested five seedlings in each watering treatment-sediment type combination (i.e., one from each plot) at each of 5 times: after 15, 30, 45, 60 and 75 days. Thus, the total experimental design included 200 lysimeters: 2 sediments × 4 treatments × 5 harvest times × 5 replications. At each harvest time, the targeted plant seedlings were carefully harvested by hand with the help of a watering hose. The roots of the sampled seedlings were carefully washed completely free of soil, taking care to maintain the integrity of the root systems. For each harvested seedling, we recorded the number of first-order roots and the presence of adventitious roots. Additionally, the maximum plant height, tap root depth and first-order root length were measured using a ruler (accurate to 1 mm), and the diameter of first-order roots and tap roots was measured with electronic Vernier calipers (accurate to 0.01 mm). We divided the harvested seedlings into aboveground parts and belowground parts, and shoot and root components were dried to a constant weight at 65°C to obtain biomass values.

### Data analysis

For each harvested *P. euphratica* seedling, we calculated the total biomass, plant height, relative weights of the roots and shoots, root-to-shoot ratio, tap root depth, number of first-order roots, total root length and total root surface area (Table 1). Prior to analysis, we transformed these variables as necessary (log, square root) after checking the assumptions of ANOVA with box plots and plots of model residuals. The experimental design was treated as a split-split-plot design with the following variables: watering treatments (fixed: 4 levels), plots (random: 4 levels nested within treatments), sediment types (fixed: 2 levels in each plot) and harvest times (fixed: 1 seedling harvested at days 15, 30, 45, 60 and 75). A partially nested ANOVA model [37] was fitted including all terms plus appropriate interactions. When interaction terms were significant, appropriate one- or two-factor ANOVA models were then fitted for each level of the other factor(s); e.g., a significant water treatment by sediment by time interaction resulted in treatment by sediment models fitted for each time separately. We applied Tukey’s tests to detect differences between treatment means after a significant main effect. All statistical analyses were conducted using SPSS software (version 11.5; SPSS, Inc., Chicago, IL, USA).

**Table 1:** Measured or calculated plant traits and allocation variables.

Variable and units	Formula
Plant height (cm)	H
Tap root depth (cm)	D
Root biomass (g)	W_R_
Shoot biomass (g)	W_S_
Total biomass (g)	W = W_R_+ W_S_
Root-to-shoot ratio	W_R_/ W_S_
Total root length (m)	L = L _tap root_ +L _first-order roots_
Total root surface area (cm^2^)	RSA = RSA _tap root_ +RSA _1st-order root_
Number of first-order roots	RNO
Relative root weight (%)	%R = (W_R_/W) × 100
Relative shoot weight (%)	%S = (W_S_/W) × 100

## Results

### Whole-plants growth responses and biomass allocation

The *P. euphratica* seedlings exhibited considerable adaptive responses to all of the watering treatments imposed during the experiment, and no seedling mortality occurred. Substantial differences in growth and biomass allocation were observed across the watering treatments and varied over time (Table 2). Sediment effects were generally significant (with the notable exception of relative weights of shoots and roots), and significant interactions were found between the watering treatments and sediment types, which also varied over time in terms of root metrics (Table 2).

**Table 2:** *F* values and significance levels from full ANOVA of the main effects and interactions in relation to the total biomass (W), plant height (H), and the relative weights of shoots (%S) and roots (%R).

Source	d.f.	H	W	%S	%R
**Treatment** ^**1**^	3	14.85***	62.21***	14.66***	14.66***
**Sediment** ^**2**^	1	10.23**	30.46***	0.00	0.00
**Treatment ×Sediment** ^**2**^	3	2.08	2.58	2.03	2.03
**Time** ^**3**^	4	185.53***	253.83***	46.54***	46.54***
**Treatment ×Time** ^**3**^	12	2.17*	24.54***	4.87***	4.87***
**Sediment ×Time** ^**4**^	4	1.05	6.31***	3.00*	3.00*
**Treatment ×Sediment ×Time** ^**4**^	12	0.75	2.10*	0.50	0.50

Superscripted numbers (1–4) indicate the error terms used in model, as follows

^1^ Plot (Treatment) with 15 d.f.

^2^ Plot (Treatment) × Sediment with 15 d.f.

^3^ Plot (Treatment) × Time with 60 d.f.

^4^ Plot (Treatment) × Sediment × Time with 60 d.f.

* *p* ≤ 0.05.

** *p* < 0.01.

*** *p* ≤ 0.001.

**Fig 1:**
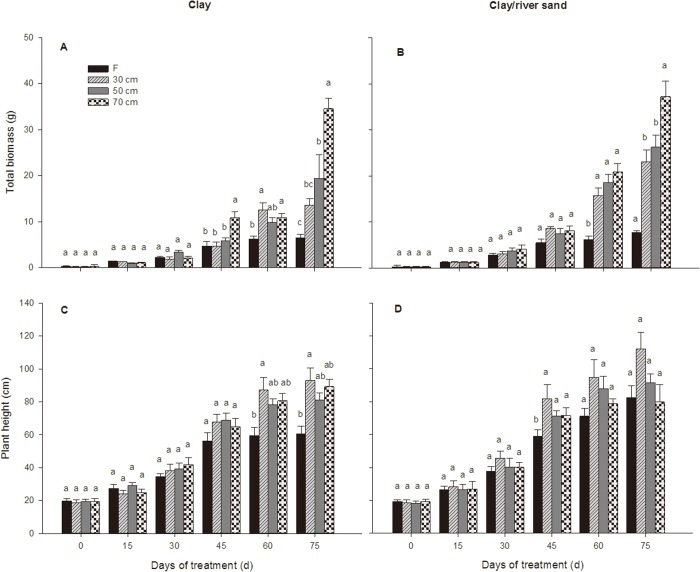
Growth-related dynamics of *P. euphratica* seedlings subjected to different treatments. Changes over time in the total biomass (A, B) and plant height (C, D) of *P. euphratica* seedlings subjected to each watering treatment in clay sediment and clay/river sediment (F = inundation treatment; 30 cm = 30 cm water table depth treatment; 50 cm = 50 cm water table depth treatment; 70 cm = 70 cm water table depth treatment. Each point represents the mean (±S.E.) value for 5 plants. The letters in the tables indicate homogeneous subsets (Tukey’s tests) at 15, 30, 45, 60 and 75 days.

At the end of the experiment, the total biomass grown under 70 cm water table depth was significantly higher than other treatments. Plant height of the seedlings grown under varying water table treatments in each sediment type were not significantly different (Fig. 1A-1D; Table 3). In addition, the total biomass of seedlings grown under a 70 cm water table was significantly greater compared with those grown under inundation, regardless of the sediment type (Fig. 1A, B); however, for seedling height, this pattern occurred only in clay sediment (Fig. 1C, D). The sediment effect was not significant for biomass, but it was for height (Table 2). While biomass increased rapidly after 45 days of growth (with the exception of inundated seedlings) (Fig. 1A, B; Table 3), height increased steadily during the experiments (Fig. 1C, D). For seedlings under varying water table conditions, the patterns between treatments varied only in seedlings in the clay sediment type, where the biomass associated with a 70 cm water table depth was significantly greater at 45 days of growth, but in the clay/river sand sediment, this pattern only occurred at the end of experiments (Fig. 1A, B; Table 3).

**Table 3:** *F* values and significance levels for the significant main effects from ANOVA of the total biomass (W), relative shoot weight (% S) and relative root weight (% R), for which there were no sediment effects, and plant height (H), separately for each harvest time interval.

	W	%S	%R	H	
	Treatment^1^			Treatment^1^	Sediment^2^
**15 days**	2.66	0.59	0.59	0.18	0.13
**30 days**	1.73	3.00 *	3.00*	0.98	0.91
**45 days**	3.89 *	1.52	1.52	3.82*	2.61
**60 days**	1.21	6.91 ***	6.91 ***	5.71**	1.71
**75 days**	4.48 **	33.75 ***	33.75***	5.28**	2.88

Superscripted numbers (1 and 2) indicate the error terms used in model, as follows

^1^ Plot (Treatment) with 15 d.f.

^2^ Plot (Treatment) × Sediment with 15 d.f.

* *p* ≤ 0.05.

** *p* < 0.01.

*** *p* ≤ 0.001.

**Fig 2:**
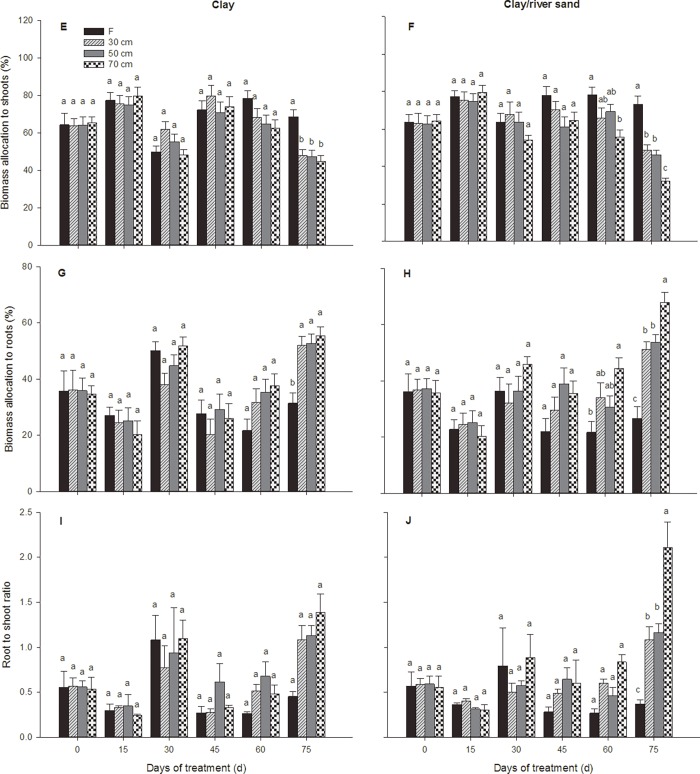
Biomass allocation dynamics of *P. euphratica* seedlings subjected to different treatments. Changes over time in the relative weights of shoots (E, F), relative weights of roots (G, H) and the root-to-shoot ratio (I, J) of *P. euphratica* seedlings subjected to each watering treatment in the clay sediment and the clay/river sediment (F = inundation treatment; 30 cm = 30 cm water table depth treatment; 50 cm = 50 cm water table depth treatment; 70 cm = 70 cm water table depth treatment. Each point represents the mean (±S.E.) value for 5 plants. The letters in the tables indicate homogeneous subsets (Tukey’s tests) at 15, 30, 45, 60 and 75 days.

Sediment type had no effect on biomass allocation (sediment effect; Table 2). *P. euphratica* seedling biomass allocation to the shoots and roots under all treatments fluctuated, showing significant variation over time (significant treatment × time interaction; Fig. 2E-2H; Table 3). Under the varying water table treatments in the clay sediment type, seedling biomass allocation to the shoots and roots did not differ significantly during the experiment, nor did it differ from that of plants under the inundation treatment after 75 days (Fig. 2E; Table 3). In the clay/river sand sediment type, the varying water table treatments only affected shoot and root biomass allocation at the final harvest (Fig. 2F; Table 3). In both sediment types, inundation significantly reduced seedling biomass partitioning to the roots at the end of the experiment, and the seedlings subjected to inundation therefore showed a significantly lower root/shoot ratio than the other three treatments (Fig. 2E-2J; Table 3). In particular, the seedlings subjected to the inundation treatment allocated less biomass to the roots than to the shoots (root-to-shoot ratio <1), while the seedlings subjected to the remaining three treatments concentrated more resources in the root system (root/shoot >1) (Fig. 2I, J). No significant effect of the 70 cm treatment on the root-to-shoot ratio was detected, although plants under the 70 cm treatment in the clay sediment tended to exhibit a higher root-to-shoot ratio than those in the other two varying water table treatments (Fig. 2I). However, this pattern was not replicated in the clay/river sand sediment, and the root-to-shoot ratios were considerably higher in the plants in the 70 cm treatment than the control plants in this sediment type at the final harvest, while the root-to-shoot ratios under the 30 cm and 50 cm treatments were equivalent (Fig. 2J).

### Adaption of root traits

**Fig 3:**
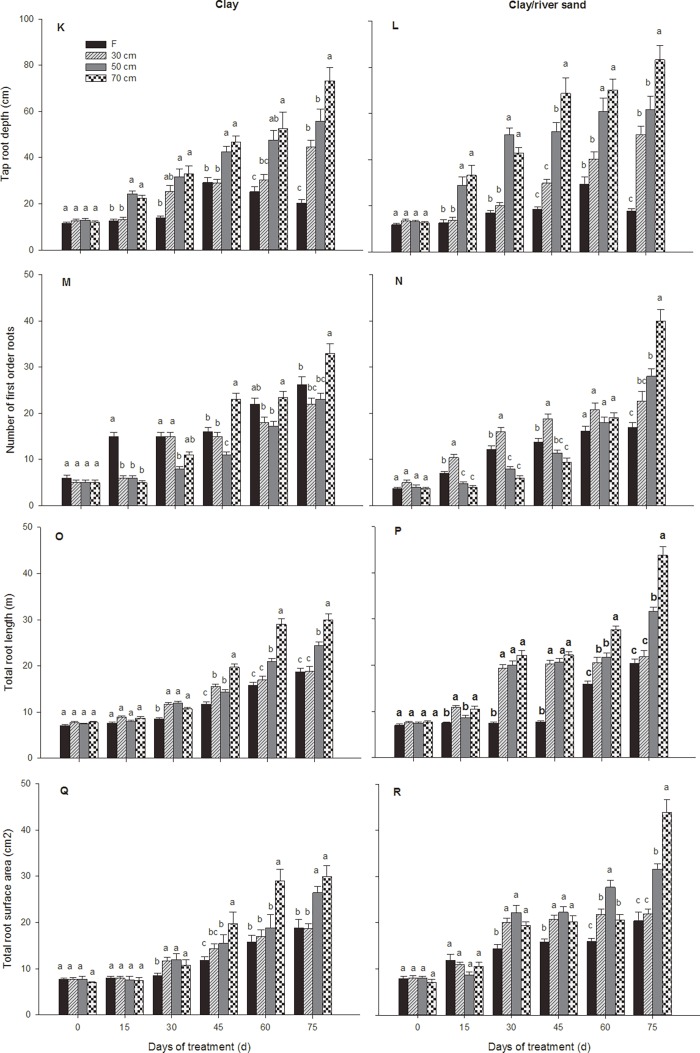
Dynamics of the root variables of *P. euphratica* seedlings subjected to different treatments. Changes over time in the tap root depth (K, L), number of 1st-order roots (M, N), total first-order root length (O, P) and first-order root surface area (Q, R) of *P. euphratica* seedlings subjected to each watering treatment in the clay sediment and the clay/river sediment (F = inundation treatment; 30 cm = 30 cm water table depth treatment; 50 cm = 50 cm water table depth treatment; 70 cm = 70 cm water table depth treatment. Each point represents the mean (±S.E.) value for 5 plants. The letters in the tables indicate homogeneous subsets (Tukey’s tests) at 15, 30, 45, 60 and 75 days.

Tap root depth was significantly affected by the sediment type, and this effect varied through time (significant sediment × time interaction; Fig. 1K, L; Table 4). A significant interaction of the treatments and sediments also affected the tap root depth (significant treatment × sediment interaction; Fig. 3K, L; Tables 4 and 5). The tap root depths in plants under the varying water table depth treatments where characterized by a rapid elongation rate throughout the growing season (Fig. 3K, L; Table 5), especially under the 70 cm treatment, where the tap root depths had increased almost six times by the end of the experiment, resulting in greater tap root depths than the control plants (Fig. 3K, L). This root elongation occurred more rapidly amongst the plants under the 70 cm treatment in the clay/river sand sediment type than amongst those in the clay-only sediment (significant sediment effect; Fig. 3K, L; Tables 4 and 5). In the inundated plants in both sediment types, tap root depths increased at a slower rate than in the control plants and were significantly shallower from 15 days onward. Moreover, tap root depths decreased after 45 days in clay sediment and after 60 days in clay/river sand sediment (Fig. 3K, L; Tables 4 and 5). The tap root depths of plants under the 50 cm treatment were significantly greater than in 30 cm treatment plants after 15 days, but the roots of the latter group grew to a comparable depth by the final harvest. In the clay/river sand sediment, tap root depths were considerably greater amongst the plants in the same water table treatments than in the control plants in the clay sediment type at the final harvest, except in the inundation treatment (Fig. 3K, L; Table 5). Adventitious roots were present by 30 days in 30% of the plants harvested from the inundation treatment. By 60 days, 60% of the plants harvested from the inundation treatment exhibited adventitious roots. These proportions remained similar or slightly lower at the final harvest.

**Table 4:** *F* values and significance levels from ANOVA of root metrics. See Table 1 for the definition of variables.

Source	d.f.	D	RNO	L	RSA
**Treatment**	3	181.67***	21.27***	122.54***	38.22 ***
**Sediment**	1	27.38***	16.47***	88.57***	81.96 ***
**Treatment × Sediment**	3	8.08***	29.57***	18.33***	2.28
**Time**	4	115.30***	357.26***	255.50***	133.77 ***
**Treatment × Time**	12	10.63***	25.61***	15.11***	9.75 ***
**Sediment × Time**	4	2.79*	3.93 *	12.90***	5.71 *
**Treatment × Sediment × Time**	12	1.82*	8.51***	7.53***	4.28 *

* *p* ≤ 0.05.

*** *p* ≤ 0.001.

**Table 5:** *F* values and significance level for the significant main effects from ANOVA of the tap root depth (D) and number of first-order roots (RNO), for which there were significant sediment effects, separately for each harvest time interval.

	D			RNO		
	Treatment^1^	Sediment^2^	Treatment^1^ × Sediment^2^	Treatment^1^	Sediment^2^	Treatment^1^ × Sediment^2^
**15 days**	28.56 ***	0.00	0.00	60.17 ***	14.63 ***	44.86 ***
**30 days**	50.60 ***	14.94 ***	8.93 ***	48.58 ***	8.13 **	9.16 ***
**45 days**	47.34 ***	5.60 *	9.01 ***	14.73 ***	29.25 ***	32.84 ***
**60 days**	26.67 ***	13.18 ***	0.84	2.96 *	3.70	5.73 **
**75 days**	61.17 ***	2.42	0.72	30.54 ***	0.52	8.20 ***

Superscripted numbers (1 and 2) indicate the error terms used in model, as follows

^1^ Plot (Treatment) with 15 d.f.

^2^ Plot (Treatment) × Sediment with 15 d.f.

* *p* ≤ 0.05.

** *p* < 0.01.

*** *p* ≤ 0.001.

Differences in all of the calculated root metrics were observed across the watering treatments and varied over time (significant treatment × time interaction Tables 4–7). The total root length and number of first-order roots in the seedlings were considerably affected by the sediment type, and this effect varied through time regarding the total root length (significant sediment × time interaction; Tables 4–6), in addition, multiple interactions also significantly affected these two root metrics (significant treatment × sediment × time interaction; Table 4). In the clay sediment, plants under the inundation treatment accumulated more first-order roots than those in the control treatments for the first 15 days of the experiment. This parameter then remained relatively constant between 15 and 45 days, after which a comparable root number to the control plants accumulated by the final harvest (Fig. 3M). In the clay/river sand sediment, the number of first-order roots in the plants under all treatments increased continuously for the first 45 days. This increase continued until the end of the experiment in the inundation treatment and the 30 cm treatment, whereas the accumulation of new roots increased rapidly in plants subjected to the 50- and 70 cm treatments after 45 days, and plants had accumulated considerably more roots under deeper water tables by the end of experiment (Fig. 3N; Table 5). In both sediment types, greater total root lengths were observed during later stages of the experiment under the 70 cm treatment (Fig. 3O, P). The total root length in plants under the 70 cm treatment remained relatively constant for the first 30 days of the experiment but then began to increase rapidly, with these roots almost tripling in length by the end of the experiment in the clay sediment type (Fig. 3O). This total root elongation occurred earlier and more rapidly amongst the 70 cm treatment plants in the clay/river sand sediment type than in the plants in the clay sediment. The total root length increased more rapidly under inundation and the 30 cm treatment after 30 days and 45 days in the clay sediment and clay/sand sediment, respectively; however, the root lengths in these treatments were still significantly shorter than in the plants under 50 cm and 70 cm treatments, regardless of the sediment type, at the final harvest (Fig. 3O, P). The effects of watering on the total root surface area varied through time (significant treatment × time interaction; Table 4). The total root surface area was also highly variable but tended to be smaller in the inundation treatments, particularly during intermediate harvests (Fig. 3Q, R; Table 7). In the clay sediment, the seedling total root surface area under the 70 cm treatment increased by a substantially greater amount than in the other treatments between 30 and 60 days but was comparable to plants under the 50 cm treatment at the end of the experiment (Fig. 3Q; Table 7). This pattern was not replicated in the clay/river sand sediment, and the total root surface area was considerably smaller in the 50 cm than the 70 cm treatments at the final harvest (Fig. 3R; Table 7).

**Table 6:** *F* values and significance levels for the significant main effects from ANOVA of the total root length (L), separately for each harvest time interval.

	L		
	Treatment^1^	Sediment^2^	Treatment^1^ × Sediment^2^
**15 days**	12.64 ***	2.29	1.61
**30 days**	40.45 ***	103.57 ***	16.09 ***
**45 days**	38.82 ***	10.31 **	12.69 ***
**60 days**	20.91 ***	0.77	13.18 ***
**75 days**	47.05 ***	30.04 ***	5.36 **

Superscripted numbers (1 and 2) indicate the error terms used in model, as follows

^1^ Plot (Treatment) with 15 d.f.

^2^ Plot (Treatment) × Sediment with 15 d.f.

* *p* ≤ 0.05.

** *p* < 0.01.

*** *p* ≤ 0.001.

**Table 7:** *F* values and significance levels for the significant main effects from ANOVA of the total root surface area (RSA), separately for each harvest time interval.

	RSA		
	Treatment^1^	Sediment^2^	Treatment^1^ × Sediment^2^
**15 days**	1.86	24.38 ***	1.25
**30 days**	10.13 ***	117.69 ***	1.40
**45 days**	7.47 ***	19.21 ***	2.22
**60 days**	10.72 ***	1.32	8.80 ***
**75 days**	23.22 ***	12.27 ***	2.59

Superscripted numbers (1 and 2) indicate the error terms used in model, as follows

^1^ Plot (Treatment) with 15 d.f.

^2^ Plot (Treatment) × Sediment with 15 d.f.

* *p* ≤ 0.05.

** *p* < 0.01.

*** *p* ≤ 0.001.

## Discussion

**Fig 4:**
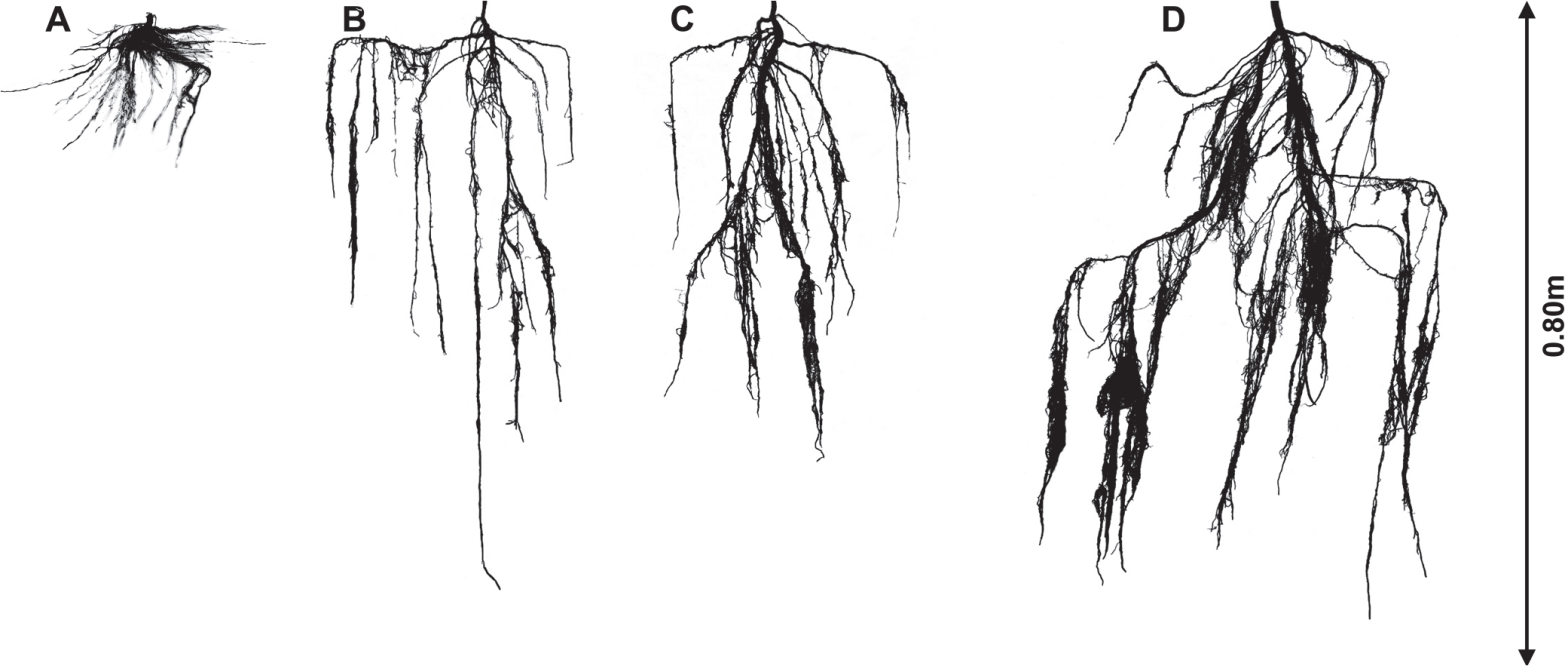
Whole root systems of *P. euphratica* grown in inundation (A), 30 (B), 50 (C) and 70 (D) cm water table depth in clay only sediment.

**Fig 5:**
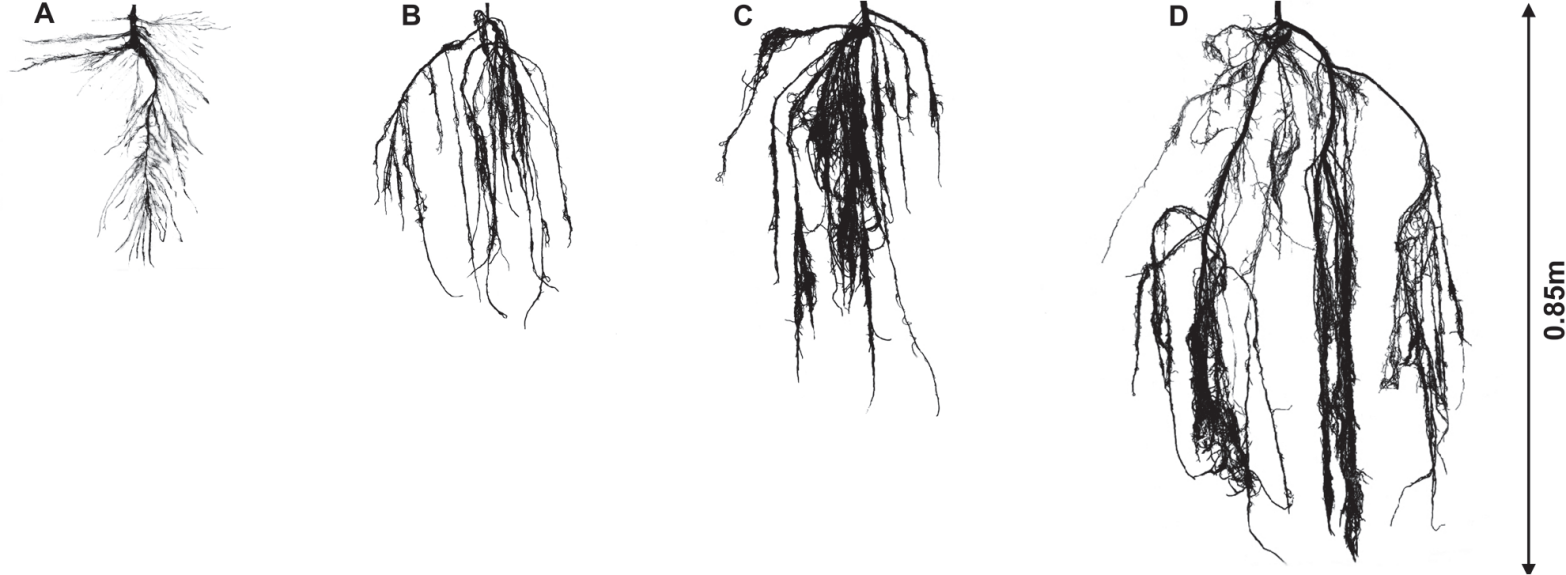
Whole root systems of *P. euphratica* grown in inundation (A), 30 (B), 50 (C) and 70 (D) cm water table depth in clay/river sand sediment.

In accordance with our hypothesis, significant plastic responses to water table depths with contrasting sediment types were observed for *P. euphratica* seedlings (Figs. 4 and 5). Furthermore, optimal partitioning theory is supported by our results, and it can be concluded that adaptive phenotypic plasticity developed or was advantageous in seedlings of this species under a range of hydrological conditions over a period of 75 days. *P. euphratica* seedlings allocated more biomass to the shoots and developed adventitious roots in adapting to inundation conditions. *P. euphratica* seedlings under deeper water table depths allocated more biomass to the roots and showed an altered root trait, with an increased total root length, suggesting that trees faced with a deeper water table are likely to increase their root length to obtain more water. Furthermore, the root systems of this species developed very different traits depending on the sediment types in which they were growing, such that even at the same water table depth, the exploitation of water by the plants will depend on both their rooting depth and the degree of root branching. Our results indicate that *P. euphratica* seedlings are capable of adapting for considerable periods under a range of hydrological conditions in different sediment types. Allocation plasticity and root plasticity both provide significant advantages for seedling growth in this desert floodplain tree species, and these effects are likely to optimize growth as a result of enhancing the chance for survival and recruitment success in riparian ecosystems.

Inundation appears to be particularly detrimental to *P. euphratica* development, although the seedlings of this species are remarkably able to maintain metabolic processes for at least 75 days under partial submergence. In both of the tested sediment types, inundation significantly reduced seedling growth in terms of most of the measured traits variables, and the rate and magnitude of the reductions tended to be linked to the duration of inundation. The responses of the *P. euphratica* seedlings to inundation were related to adaptive phenotypic plasticity in biomass allocation and most of the root traits we examined. Biomass partitioning between the shoots and roots varied in response to increasing durations of inundation, in accord with the predictions of optimal partitioning theory [23]. We observed the development of adventitious roots in response to inundation, as occurs in some flood-tolerant Cruciferae species, e.g., *Lepidium latifolium* [23]. Nevertheless, while adventitious roots were recorded in plants in the inundation treatments, this was not a consistent response. Adventitious rooting is one of the important adaptive mechanisms of wetland plants for replacing existing roots that have been killed or whose function is impaired by anaerobic environments [23, 38]. This finding may partially explain why seedlings were still able to maintain metabolic processes when their tap roots rotted in the inundation environment during later stages of the experiment. Moreover, lateral branching of roots, which might promote plant stability in the face of flood scouring [23, 38], could also present advantages, as tap root depths did not increase correspondingly. Other morphological or physiological mechanisms that were not considered here, such as aerenchyma development in the roots [39], may contribute to the ability of *P. euphratica* seedlings to persist through long periods of inundation, and some of these mechanisms may exhibit adaptive plasticity. To a large extent, it appears that *P. euphratica* seedlings adjust to inundation through adaptive phenotypic plasticity and allocational plasticity, thereby reducing their resource requirements and minimizing damage under saturated conditions, such as the accumulation of toxic materials in saturated soils [15, 38]. Therefore, our results confirm our hypothesis that adaptive phenotypic plasticity is likely to develop or be advantageous in the seedlings of *P. euphratica* under inundation condition.

*P. euphratica* seedlings appear to be considerably more favor of the 70 cm treatment than inundation. The development of seedlings subjected to the 70 cm treatment applied in this experiment did not differ from that of control plants until approximately 60 days, despite substantial differences in the application of water. After this time, however, the increases in biomass and total root length accelerated significantly in the plants under the 70 cm treatment. In the clay/river sand sediment, water acquisition in *P. euphratica* seedlings may be attributed to the tendency of plants to enlarge their root surface area in the 70 cm treatment, initially by increasing the accumulation of new roots and root length and subsequently, following an increasing duration of water demand, through expansion of the absorptive root surface. The plasticity of plants in terms of enlarging their surface area in response to lower water resources is a well-known response [40] and may be considered adaptive, as it enables plants to enhance water acquisition [41]. However, the total root surface area did not increase with the total root length in the clay sediment, suggesting that seedlings faced with deeper water tables are not always likely to increase their root surface area to obtain more water [9]. This result may have been due to the increased capillary ascension associated with finer sediment. We detect significantly greater elongation of *P. euphratica* roots in response to a deeper water table depth, as has been recorded in seedlings of some drought-tolerant riparian species, such as *Populus alba* [9], *P. euphratica* and *Tamarix ramosissima* [27]. Moreover, the root systems of *P. euphratica* developed very different traits in the different sediment types under the same hydrological conditions. In general, branching will be greater in clay sediment and where water tables are shallower, whereas downward growth will be greater in the clay/river sand sediment and where water tables are deep. These observations are in good agreement with the findings of a study by Hughes et al. (1998) [42], who investigated cuttings of male and female black poplar grown in different sediment types with different water table levels.

Recent theory [11, 18, 43] predicts that true adaptive plasticity is likely to evolve or be beneficial in variable environments. Our finding that *P. euphratica* seedlings demonstrated great phenotypic plasticity when growing under a range of hydrological conditions in different sediment types is consistent with this hypothesis. In response to a lower water table depth, *P. euphratica* seedlings appear to exhibit phenotypic plasticity in the form of increased development of new roots and accelerated root production or expansion. In clay/river sand sediment, because the resulting increases in root length enable the root surface area to be enlarged, this phenotypic variation can be perceived as adaptive. These adaptations of the root architecture of seedlings suggest that the plants are searching for deep water reservoirs and intensely exploiting these resources [9, 44]. High biomass allocation to roots is often related to higher survival rates through improved water acquisition [45, 46], linked to reaching higher-moisture soil layers and exploring larger soil volumes [27, 41]. On the other hand, an extended period of inundation greatly reduced growth and inhibited the increase in the root-to-shoot ratio observed in the control plants. Nevertheless, it may be less risky and costly for seedlings to adopt altered phenotypes, thereby allowing plants to survive, than to die during inundation. This hypothesis is consistent with the observation that some flood-tolerant species typically adjust their biomass allocation patterns by devoting more biomass to the shoots to acquire oxygen and reducing biomass allocation to the roots to reduce oxygen demand [23, 47]. *P. euphratica* appears to be a plant that exhibits plasticity that is appropriate for, or at least allows it to survive in saturated conditions, even though it may not grow well under these conditions. This plasticity may constitute an adaptation to arid or semiarid riparian habitats, and such adaptive phenotypic plasticity is likely to have developed or be advantageous in seedlings of this species, as the changes in the environmental conditions it experiences are highly variable in their timing and duration. Thus, investing in plastic responses to inundation and to a range of hydrological conditions may be more advantageous than following relatively fixed developmental trajectories.

Although *P. euphratica* seedlings exhibit considerable adaptability to both inundation and a gradient of water table depths, the effects on seedling establishment in the field are likely to be exacerbated by additional pressures such as interspecific competition. In a study on seedling competition between native *Populus deltoides* and exotic *Tamarix ramosissima* across water regimes and sediment types in the southwestern United States, Sher et al. (2003) found that stream flow management that promotes *Populus* establishment could also facilitate the control of *Tamarix* invasions across a range of substrates, and *Populus* may exhibit the greatest competitive superiority in a wet environment where there is a healthy draw-down of the water table [35]. Li et al. (2013) also reported that recent water table declines along the Tarim River could facilitate the establishment of *T. ramosissima*, as *T. ramosissima* responds more rapidly than *P. euphratica* to altered water table availability. Thus, *Populus* might be under threat of losing its dominant status along the riparian corridor of the Tarim River, where hydrological conditions have been altered intensively in recent decades [27]. Water table dynamics are likely to be another key reason for the competitive success of *Populus* species [35]. Alteration of the rivers in the middle reach of the Tarim River with dykes has been cited as the predominant cause of declining *Populus* seedling establishment [28, 35]. It appears that river alterations can pose a threat to *Populus* species, not only when there are no overbank floods and/or water tables recede too quickly, but also when such changes result in a stagnant water table. Decreased river flows can lead to less deposition of large-particle sediments during inundation events [35]. Declining flooding frequencies in the middle and lower reaches of the Tarim River, for instance, could result in reduced opportunities for *Populus* seedling establishment and the subsequent domination of these areas by *Populus* trees. Similar patterns have been documented for other species in floodplains where flow regimes have been heavily modified, e.g., the invasion of floodplain by *T. ramosissima* in Socorro, New Mexico, United States [35]. Given the potential ecological consequences of such shifts in floodplain vegetation communities, further research is warranted to more precisely establish the conditions under which *Populus* seedling establishment occurs.

## Conclusions

*P. euphratica* seedlings grown in both clay and sandy sediments exhibited considerable adaptability to inundation and varying water table depths in our experiment. Sediment effects were generally significant (with the notable exception of relative weights of shoots and roots), and significant interactions were found between the watering treatments and sediment types; these effects varied over time for the examined root metrics. In general, the root systems of *P. euphratica* developed very different traits in the different sediment types under the same hydrological conditions. At the whole-plant level, *P. euphratica* reduced root biomass allocation and exhibited adaptive mechanisms in response to the inundation environment involving the development of adventitious roots. *P. euphratica* allocated more biomass to the roots and displayed an altered root trait. Our results demonstrated that *P. euphratica* seedlings adapt to a wider range of hydrological conditions in different sediment types through phenotypic plasticity and allocation plasticity responses. Thus, adaptive plasticity should promote establishment and persistence in a new environment.

## Supporting Information

S1 Dataset Original data. (XLSX)
